# Adenoma detection rates in an opportunistic screening colonoscopy program in Iran, a country with rising colorectal cancer incidence

**DOI:** 10.1186/s12876-014-0196-8

**Published:** 2014-11-18

**Authors:** Alireza Delavari, Faraz Bishehsari, Hamideh Salimzadeh, Pejman Khosravi, Farnaz Delavari, Siavosh Nasseri-Moghaddam, Shahin Merat, Reza Ansari, Homayoon Vahedi, Bijan Shahbazkhani, Mehdi Saberifiroozi, Masoud Sotoudeh, Reza Malekzadeh

**Affiliations:** Digestive Oncology Research Center, Digestive Disease Research Institute, Shariati Hospital, Tehran University of Medical Sciences, Tehran, Iran; Sasan Alborz Biomedical Research Center, Masoud Gastroenterology and Hepatology Clinic, Tehran, Iran; Department of Internal Medicine, Division of Gastroenterology, Rush University Medical Center, Chicago, IL USA; Non-Communicable Disease Research Center, Tehran University of Medical Sciences, Tehran, Iran

**Keywords:** Screening colonoscopy, Colonic polyps, Colon cancers

## Abstract

**Background:**

Data on the quality of colonoscopies in populations with rising colorectal cancer (CRC) incidence is scarce. We aimed to calculate the adenoma detection rates (ADR), and assess the quality of colonoscopies in an opportunistic screening colonoscopy program in Iran.

**Methods:**

All the colonoscopy and pathology reports of asymptomatic adults over age 50 who underwent screening colonoscopy between June 2007 and March 2013 were reviewed. The colonoscopy quality indicators including ADR were calculated, and patient factors associated with the adenoma detection were determined.

**Results:**

A total of 713 asymptomatic adults aged 50 years and older who underwent their first-time screening colonoscopy were included in this study. ADR and advanced-ADR were 33.00% (95% CI: 29.52-36.54) and 13.18% (95% CI: 10.79-15.90), respectively. We observed a significantly higher rate of cecal intubation in patients with fair or better bowel preparation compared to those with poor prep, 90.00% *vs.* 70.45%, respectively (*P* < 0.001). Bowel preparation (adjusted OR: 2.49, 95% CI: 1.75-3.55), older age (≥60) (adjusted OR: 1.70, 95% CI: 1.22-2.36), and male gender (adjusted OR: 1.39, 95% CI: 1.01-1.92) were associated with the adenoma detection.

**Conclusions:**

Our ADR in both genders meets and exceeds the recommended colonoscopy quality benchmarks. The polyp and adenoma detection rates in the current study are comparable to those reported from Western countries where the incidence of CRC is traditionally high. These data are in line with the epidemiologic transition of CRC in Iran.

## Background

Colorectal cancer (CRC) is the third most commonly diagnosed cancer in the world [1]. It is estimated that the global CRC rates will double over the next two decades in the light of an ongoing rise of the disease’s incidence in the developing countries [2]. This calls for a need to develop preventive strategies for CRC in these populations. Colonoscopy is an accepted modality for CRC screening among average-risk patients [3,4]. In Western countries with traditionally high incidence of CRC, implementation of screening colonoscopies with polypectomies in average-risk individuals have led to reductions in the incidence and mortality of the disease [5,6]. Therefore, screening colonoscopy is potentially an efficacious preventive method to combat the global rise of CRC. However, developing countries that are usually equipped with fewer resources, may view organized colonoscopy as a public health burden. Therefore, prior to consideration of a mass screening colonoscopy, the quality of the current practice of colonoscopy, and factors associated with its quality should be first identified and optimized. Among several colonoscopy quality indicators, adenoma detection rate (ADR) has been the most widely used metric for benchmark colonoscopy quality [7,8].

Iran, similar to several other developing countries, is experiencing a significant rise in the incidence of CRC over the recent decades [9,10]. Although mass screening of CRC is not yet available, opportunistic screening colonoscopy is performed mostly by gastroenterologists both in public and private medical centers. Screened patients are either self-referral or referred from physicians’ offices. Here, for the first time in the region, we aim to assess the quality of screening colonoscopy among average-risk adults in a private referral gastrointestinal center in Tehran, Iran. We measured ADR, and determined patient factors associated with the adenoma detection in our practice.

## Methods

### Study design

We retrospectively reviewed screening colonoscopy and the corresponding pathology reports at Masoud Clinic, a referral private center for gastrointestinal diseases in Tehran, Iran. All study materials and procedures were approved by the Institutional Review Board of Digestive Disease Research Institute, Tehran University of Medical Sciences.

### Patients and procedures

All asymptomatic individuals aged 50 years and older who underwent their first-time screening colonoscopy between June 2007 and March 2013 were included in the current study. Thirteen endoscopists performed 713 colonoscopy examinations under conscious sedation with a combination of Pethidine and Midazolam using high image resolution colonoscopes (Olympus CF*-*Q160L and Pentax EC-3890LK). Patients used three Bisacodyl tablets and 4 L of Polyethylene Glycol in divided doses for bowel preparation on the day before colonoscopy. Characteristics of colorectal polyps (i.e., number, size, form, and location) were documented in the colonoscopy reports by gastroenterologists. Detected polyps were removed during colonoscopy and the specimens were transferred in separate formalin containing jars to the pathology department. Two experienced gastrointestinal pathologists evaluated the specimens and entered the histological features of polyps in a pathology database. Written informed consent was obtained from the patients.

### Measurements and definitions

We retrieved data on demographic characteristics of the patients, quality of colon preparation, complications, and the rate of successful cecal intubation. Data on number, size, and other features of colorectal lesions were classified by location: the distal colon was identified as the rectum through the splenic flexure, and proximal colon was defined as anywhere between distal transverse through the cecum. Data on duration for each colonoscopy and withdrawal time were not available from the colonoscopy reports.

The quality of bowel preparation was rated using the Aronchick categorical scale (i.e., excellent, good, fair, poor, and unsatisfactory) [11]. Histopathology of all colorectal lesions was documented according to the World Health Organization criteria as follows: hyperplastic, serrated, tubular, tubular-villous, villous, and cancer [12]. Serrated lesions were grouped as: hyperplastic polyps, sessile serrated adenomas/polyps and traditional serrated adenomas. Polyps with feature of tubular, tubular-villous, villous were defined as adenoma. Advanced adenomas included those that were ≥10 mm in size, had villous or tubular-villous histology, or high-grade dysplasia.

PDR, ADR, and advanced-ADR were defined as the number of procedures with at least one polyp, adenoma, and advanced-adenoma detected divided by the total number of procedures performed, respectively.

In order to determine whether the volume of colonoscopies performed by each endoscopist could affect the quality indicators, we eliminated part-time endoscopists who had performed <40 colonoscopies during the study period. For this analysis, full-time endoscopists who performed more than 40 colonoscopies (n = 4) were included and grouped into high (>100 procedures, n = 2) *vs*. low (40–100 procedures, n = 2) volume groups, and their detection rates were compared.

### Statistical analysis

We had a full access to all the colonoscopy reports as well as the pathology files of Masoud clinic within the study time. Pathology reports were identified upon manual review of the patients’ records. Pathology data was linked with the colonoscopy reports to ensure avoidance of any data duplications. In total, 822 colonoscopy reports were reviewed. Hundred-nine cases, whose pathology reports were not available as their specimens were sent elsewhere for the pathology review, were excluded. Therefore 713 cases with available pathology information were included for the final analysis and calculation of the studied quality indicators. We also excluded symptomatic patients, and individuals who are considered high-risk for CRC with a familial pattern of the disease (i.e., CRC in the first degree relatives, heredity nonpolyposis CRC, and familial adenomatous polyposis).

Continuous data were presented as means with 95% confidence interval (CI). Categorical variables were reported as number and proportions with 95% CIs. The overall estimates for PDR, ADR, and advanced-ADR were calculated using patient-level data. Univariate and multivariate analysis by logistic regression model were used at the patient level data to identify patient factors associated with presence of at least one adenoma per colonoscopy. Rates and proportions were compared between sub-groups using the chi-square test or the Fisher’s exact test, where appropriate. For statistical significance we considered a *P* value smaller than 0.05 applying 2-tailed statistical tests. Statistical analyses were performed using Stata/MP software, version 11.

## Results

### Demographics and colonoscopy data

Seven hundred thirteen (713) asymptomatic adults aged 50 years and older who underwent their first-time screening colonoscopy were included in this study. The mean age of the screened individuals was 61.68 (95% CI: 61.06 - 62.30) years, with a relatively equal proportion of both genders including 53% (n = 380) males, and 47% (n = 333) females. The quality of bowel preparation was excellent to fair in 62.97% (n = 449) of colonoscopies. Cecal intubation was documented in 82.75% (n = 590) of patients (Table 1). There were no serious complications (i.e., perforation, significant hemorrhages) or deaths associated with the screening colonoscopies.

**Table 1:** **Patient characteristics and colonoscopy data**

	**All (n = 713)**
Mean age, years (95% CI)	61.68 (61.06-62.30)
Gender Male/Female, n (%)	380 (53.30)/333 (46.70)
Preparation quality, n (%)	
Excellent/Good/Fair	449 (62.97)
Poor/Unsatisfactory	264 (37.03)
Scope progress, n (%)	
Cecal intubation	590 (82.75)
Incomplete	123 (17.25)

CI, confidence interval.

### Detection rates

The quality indicators are shown in Table 2. Overall, 521 polyps (in 259 individuals) were resected during the colonoscopy procedures. Our calculated PDR was 36.33% (95% CI: 32.79-40.00). On the basis of the pathology data, serrated lesions were reported in 8.27% (95% CI: 6.36-10.54, n = 59) of all cases with subtype detection rates of 7.01% (95% CI: 5.25-9.14, n = 50) for hyperplastic polyps and 1.54% (95% CI: 0.77-2.74, n = 11) for sessile serrated polyps. In all, 33.00% (95% CI: 29.52-36.54, n = 235) of patients had at least one adenoma (ADR), and 13.18% (95% CI: 10.79-15.90, n = 94) had at least one advanced adenoma in their exam; 0.84% (95% CI: 0.30-1.82, n = 6) of patients were diagnosed with carcinoma (Table 2).

**Table 2:** **Detection rates of colonoscopic lesions per patient**

	**All (n = 713)**
Polyps, n (%)	259 (36.33)
Hyperplastic polyps, n (%)	50 (7.01)
Sessile serrated polyps, n (%)	11 (1.54)
Adenomas, n (%)	235 (33.00)
Advanced adenomas, n (%)	94 (13.18)
Invasive carcinomas, n (%)	6 (0.84)

### Patient factors associated with adenoma detection

Male gender was associated with higher chance of detection of at least one adenoma (36.58, 95% CI: 31.73-41.64) as compared to women (28.83%, 95% CI: 24.02-34.02) (*P* = 0.028). In addition to the male gender, older age (60 and older) as well as fair or better bowel preparation (excellent/good/fair bowel prep) were significantly associated with adenoma detection in univariate analysis (both *P* < 0.05) (Table 3).

**Table 3:** **Results of univariate analysis for factors associated with adenomas (n = 713)**

	**Adenoma detection rate**	***P***
	no* (percentage, 95% CI)	
Gender		
Female	96/333 (28.83, 24.02-34.02)	
Male	139/380 (36.58, 31.73-41.64)	0.028
Age		
<60	83/307 (27.03, 22.15-32.37)	
≥60	152/406 (37.44, 32.71-42.35)	0.003
Bowel preparation		
Poor/Unsatisfactory	57/264 (21.59, 16.78-27.05)	
Excellent/Good/Fair	178/449 (39.64, 35.10-44.36)	<0.001

*Values are number of patients with adenoma/total number of patients; patients without adenoma serve as the reference group; CI, confidence interval.

Results of multivariate analysis showed that fair or better bowel preparation was the strongest predictor of adenoma detection (adjusted OR: 2.49, 95% CI: 1.75-3.55, *P* < 0.001). Male gender (adjusted OR: 1.39, 95% CI: 1.01-1.92, *P* = 0.046) and older age (≥60) (adjusted OR: 1.70, 95% CI: 1.22-2.36, *P* = 0.002) remained predictors of adenoma detection in the multivariate analysis (Table 4). These results were not different when only procedures with successful cecal intubation (n = 590) were included (Data not shown).

**Table 4:** **Results of logistic regression analysis with adenomas as dependent variable (n = 713)**

	**Adjusted odd ratio (95% CI)**	***P***
Gender (male *vs*. female)	1.39 (1.01-1.92)	0.046
Age (≥60 *vs*. <60)	1.70 (1.22-2.36)	0.002
Bowel preparation (excellent-to-fair *vs*. poor-to-unsatisfactory)	2.49 (1.75-3.55)	<0.001

CI, confidence interval.

High-volume endoscopists who made up about 70% (n = 489) of the total procedural volume during the study period, had an average ADR of 34.56% (95% CI, 30.35-39.00), which was significantly higher than the average ADR (20.00%, 95% CI, 12.66-29.18) achieved by the low-volume group (p = 0.004). Although, higher-volume endoscopists also tended to have a higher detection rate of advanced adenomas (12.88%, 95% CI: 10.04-16.18), in comparison to the low-volume group (7.00%, 95% CI: 2.86-13.90), this difference did not reach significance (p = 0.09).

### Bowel preparation and quality measures

The quality of bowel preparation was fair or better in 62.97% (n = 449) compared to poor in 37.03% (n = 264) of colonoscopies. Patient-related factors (e.g., age and gender) were not statistically different between the two groups (Data not shown). However we observed a significantly higher rate of cecal intubation in patients with fair or better bowel preparation compared to those with poor preparation, 90.00% (404/449) *vs.* 70.45% (186/264), respectively (*P* < 0.001). Moreover, ADR in the former group (39.64, 95% CI: 35.10-44.36) was nearly twice as high compared to the poor preparation group (21.59, 95% CI: 16.78-27.05) (*P* < 0.001).

The rate of cecal intubation adjusted for bowel preparation, varied from 71.00% to 95.00% among the endoscopists who performed ≥40 colonoscopies during the study period.

## Discussion

Adenoma detection rate has been shown to correlate with reduced rates of interval CRC [4], and is a validated metric for the quality assessment of screening colonoscopy programs [7,8]. Our study on average-risk individuals from an opportunistic screening setting in Iran indicates an ADR of 33%, corresponding to a PDR of 36.33%. These rates are in line with previous reports from the screening settings in populations that are considered to have high-incidence of CRCs [13-15]. We found an average-risk ADR of 36.58% in men and 28.83% in women, both higher than the minimum benchmarks (ADR of 25% for men and 15% for women) recommended by the United States Multi-Society Task Force for screening colonoscopies [7,16]. The advanced-ADR in the current series was 13.18%, which exceeded the rates (5 to 10%) suggested by Coriat R et al., and Ferlitsch M et al., although there is no threshold value for advanced-ADR that is currently recommended by the guidelines [17,18].

The limitations of the current study were mostly embedded in its retrospective design and a relatively small sample size. Moreover, data on withdrawal time, another well-established colonoscopy quality indicator, was not available in our colonoscopy reports. These all limited our ability to analyze the effect of endoscopist factors on the adenoma detection rates.

Patient factors affecting the adenoma detection in the current series included bowel preparation, gender and age. In our multivariate analysis, fair or better bowel preparation remained the strongest predictor for the adenoma detection, which is consistent with the previously published series [19,20]. In contrast, an inadequate bowel preparation is known to increase the rate of missed polyps [21]. The relatively high rate (~37%) of poor bowel preparation could be due to knowledge deficiencies and insufficient compliance with the prep regimens among our patients [22]. In our series, verbal instruction was the common approach taken by the endoscopist physicians and/or medical staff to provide patients with the information on the bowel prep prior to the procedure. This method of communication seems not to be efficient as suggested by the high rate of poorly prepped patients in our series. In fact, it has been shown that counseling sessions and written instructions outlining the methods and rationale for bowel preparation before colonoscopy can optimize colonoscopy quality [23,24]. Therefore, in our screening colonoscopy programs, it is advisable to involve the medical staff in patients’ education prior to their procedures in order to increase the compliance with the bowl regimen, which could lead to an increase in the quality of our colonoscopies, and eventually the ADRs.

Other predictors of adenoma detection in our study, namely older age and male gender are in agreement with the results of the prior reports [18,25-27]. Overall we had an 83% cecal intubation rate which is lower than the recommended threshold of ≥95% for screening colonoscopy [3,28]. Our lower cecal intubation rate, at least in part, could be due to a relatively high percentage of individuals with poor bowel preparation in our series. In support of this explanation, the cecal intubation rate was significantly higher in patients who had fair or better bowel preparation (90%) compared to those with poor preparation (~70%). However the cecal intubation rate of 90% is still lower than the quality benchmark (≥95%) [3,28] which could indicate areas in technical performance that need improvement. Further studies are warranted to elucidate whether an overall lower cecal intubation rate in our series, could be mainly operator or procedure (related to sedation, or difficulty of the procedure) dependent.

We observed an overall remarkable rate of the advanced adenoma lesions (13.18%) in our series. This could be explained by presence of good detectors of advanced lesions, and/or the characteristics of our series. To support the former explanation, we found that higher-volume endoscopists who performed the majority of the procedures, tended to have a higher detection rate of advanced adenomas. Furthermore, we observed an upward trend for presence of advanced-ADR across age-groups ranging from 7.64% (95% CI, 4.01-13.00) in 50–54 year old patients to 17.54% (95% CI, 12.84-23.11) in patients aged 65 years or older (p = 0.005). Of note, almost third of the screened individuals (32%) belonged to the latter group, which could potentially contribute to the high advanced adenomas in our series. Therefore, combination of good detection of advanced lesions by the high volume endoscopists, and presence of relatively high proportion of older individuals who had higher rates of advanced lesions can account for the observed rate of advanced-ADR in our study.

Number of individuals (n = 822) who underwent screening colonoscopy over the study period, reflects that the actual uptake of screening colonoscopy is presumably low in Iran. Beside the lack of organized screening, several potential barriers to CRC screening exists in Iranians’ socio-cultural content which have been identified by our group and could potentially decrease the colonoscopy uptake. Among these factors are lack of awareness about colon cancer and available screening tests, being asymptomatic, screening not being recommended by the primary care physicians, and finally sense of fear, wrong beliefs and misconceptions about cancer prevention [29]. Therefore the screening barriers should be addressed via targeted/tailored educational efforts to increase its uptake, before implementing an organized CRC screening program in the country.

To our knowledge, the current study is the first report on measuring quality indicators in a screening colonoscopy program in Iran, a country that is experiencing a rise in the incidence of CRC. It includes average-risk adult individuals who were examined consecutively in a well-equipped referral gastroenterology clinic, and therefore the calculated ADR is more likely to be close to the actual rates in average-risk population in Iran. The series also encompasses comparable numbers of each gender, which allowed us to measure the quality indicators separately for each sex. Further studies with larger sample size and inclusion of the procedure withdrawal time would be needed to better evaluate the quality of screening colonoscopies in Iran.

## Conclusions

In summary, this single-center study highlights the feasibility of an opportunistic screening colonoscopy program in Iran. Our polyp and adenoma detection rates are comparable to those reported from Western countries with high CRC rates, and are in line with the observed epidemiologic transition of CRC in Iran.

## Abbreviations

CRC: Colorectal cancer
ADR: Adenoma detection rate
PDR: Polyp detection rate
CI: Confidence interval
